# Longitudinal assessment of changes in muscle composition using proton density fat fraction and T2* in patients with and without incidental vertebral compression fractures

**DOI:** 10.3389/fendo.2025.1549068

**Published:** 2025-03-03

**Authors:** Yannick Stohldreier, Yannik Leonhardt, Jannik Ketschau, Florian T. Gassert, Marcus R. Makowski, Jan S. Kirschke, Georg C. Feuerriegel, Philipp Braun, Benedikt J. Schwaiger, Dimitrios C. Karampinos, Nina Hesse, Alexandra S. Gersing

**Affiliations:** ^1^ Department of Neuroradiology, Ludwig Maximilians University Hospital, Ludwig Maximilians University (LMU) Munich, Munich, Germany; ^2^ Department of Diagnostic and Interventional Radiology, Klinikum Rechts Der Isar, School of Medicine, Technical University of Munich, Munich, Germany; ^3^ Department of Neuroradiology, Klinikum Rechts Der Isar, School of Medicine, Technical University of Munich, Munich, Germany; ^4^ Department of Radiology, Ludwig Maximilians University Hospital, Ludwig Maximilians University (LMU) Munich, Munich, Germany

**Keywords:** incidental vertebral compression fractures, magnetic resonance imaging, muscle, spine, proton density fat fraction, chemical shift encoded MRI, bone mineral density

## Abstract

**Objective:**

Chemical shift encoded-based water-fat separation magnetic resonance imaging (CSE-MRI) is an emerging noninvasive tool for the assessment of bone and muscle composition. This study aims to examine both the predictive value and the longitudinal change of proton density fat fraction (PDFF) and T2* in the paraspinal muscles (PSM) in patients with and without the development of an incidental vertebral compression fracture (VCFs) after 6 months of follow-up.

**Methods:**

Patients (N=56) with CT and 3T CSE-MRI of the lumbar spine at baseline and CSE-MRI at 6 months follow-up were included in this retrospective study. Patients who, on average, developed an incidental VCF one year after baseline MRI (VCF: N=14, 9 males, 66.8 ± 7.9 years) were frequency matched by age and sex to patients without VCFs (non-VCF) at baseline and follow-up (non-VCF: N=42, 27 males, 64.6 ± 13.3 years). Mean PDFF, T2*, and cross-sectional area (CSA) values from the autochthonous PSM of the thoracolumbar spine (T11-L4) and opportunistic CT-based bone mineral density (BMD) measurements were obtained for each individual. The associations between baseline measurements, longitudinal changes in PDFF, T2*, CSA of the PSM and the occurrence of VCFs at follow-up were evaluated using linear and logistic multivariable regression models. ROC analyses were used to assess cutoff values for predicting the development of VCFs.

**Results:**

No significant difference in PDFF of the PSM was found between the VCF and non-VCF group at baseline (VCF/non-VCF 8.5 ± 13.8% vs. 5.0 ± 4.6%; p=0.53). In multivariable linear regression models adjusted for sex, age and baseline BMD, PDFF values of the PSM increased significantly over 6 months in the VCF group (2.4 ± 2.8% vs. -1.0 ± 2.3%, p<0.001), while T2* values of the PSM showed a significant decrease (p ≤ 0.01). ROC analyses identified a PDFF increase of 0.2% in the PSM as the optimal cutoff value to distinguish between patients with and without VCF (AUC 0.86, 95% CI [0.74-0.98], p<0.001).

**Conclusion:**

Longitudinal PDFF-based assessment of the PSM composition may be a useful indicator for the prediction of the development of vertebral compression fractures.

## 1 Introduction

In our aging society, osteoporosis is a significant health issue with insufficiency fractures of vertebrae being linked to increased mortality rates and a substantial economic burden (1–4). Early detection and reliable assessment of osteoporosis are crucial for preventing vertebral fractures. Dual-energy x-ray absorptiometry (DXA) and quantitative CT (qCT) are currently the diagnostic reference standard for the assessment of osteoporosis. However, qCT offers more precise bone mineral density (BMD) measurements compared to DXA, while at the same time resulting in higher radiation exposure of the patient (5). Reduced BMD is the major risk factor for incidental vertebral compression fractures (VCF) (6). Osteoporosis and bone loss are closely linked to sarcopenia and poor muscle strength through endocrine pathways (7). Both are independently established risk factors for vertebral fractures (8–11). Bone marrow is composed of various cell types within a trabecular bone matrix and its composition is influenced by several metabolic and external factors (12). Increased fat content is part of the pathophysiology of osteoporosis in the spine and is driven by enhanced proliferation of mesenchymal stem cells into adipocytes (13–15). A radiation free approach for fat quantification is proton density fat fraction (PDFF) mapping via chemical-shift encoding-based water-fat MRI (CSE-MRI) (16). The PDFF technique has shown to be a reliable measurement tool for fat quantification in different tissues and several previous studies have demonstrated a negative correlation between the BMD and PDFF of the vertebral bone marrow (17–24). The advantage of CSE-MRI over spatially limited measurement methods, such as single voxel proton magnetic resonance spectroscopy (MRS), is that the heterogeneous composition of the bone marrow and PSM as well as the cross-sectional area (CSA) of the PSM can be assessed simultaneously. In clinical practice, CSE-MRI can easily be integrated into routine MR imaging protocols. A recent study reported an increase of PDFF over one year within the vertebral bodies before an incidental fracture occurred while the BMD remained unchanged, indicating that PDFF could be used as a predictive biomarker for bone health (25). Fat infiltration may be influenced by external stress factors, such as myelotoxic chemotherapy, or by metabolic disorders like diabetes (26–28). Additionally, a correlation of fat infiltration of the vertebral bone marrow and the paraspinal muscles (PSM) was previously reported (29). CT- and MRI-based fat quantifications in previous studies have also demonstrated an inverse correlation between fat infiltration of the PSM and reduced muscle strength as well as spinal instability (30, 31). CSE-MRI also allows for the assessment of T2*. In vertebral bodies, T2* is linked to the osseous microarchitecture, showing an inverse correlation with BMD (29, 32).

The aim of this study is to investigate the relationship between the occurrence of vertebral compression fractures (VCF) and PDFF and T2* measurements of the PSM and the vertebral bone marrow in the lumbar region.

## 2 Materials and methods

### 2.1 Study design and patient selection

The study was approved by the local institutional review committee (Ethics Commission of the Medical Faculty, Technical University of Munich, Germany; Ethics proposal number 2022-433-S-SR). All patients gave written and informed consent prior to their participation in the study according to the Declaration of Helsinki. Between January 2018 and June 2021, a total of 200 patients underwent MRI protocol of the abdomen including a multi-echo gradient-echo sequence of the thoracolumbar spine and a CT including the lumbar spine from which opportunistic BMD measurements were derived as part of their clinical routine (33, 34). Exclusion criteria for MR imaging included pregnancy, presence of metal implants, and general contraindications for MR imaging (e.g., pacemakers). The 6 months follow-up CSE-MR images of all these patients were screened for newly detected vertebral compression fractures, which had not been present in the previous MR and CT scans. Patients with an incidental vertebral compression fracture (VCF) were retrospectively included, provided the fracture showed no signs of malignancy (e.g., osseous metastasis) or was caused by high-energy trauma. In total, 14 patients met all criteria and were enrolled in our study. These patients were frequency matched for age and sex in a 1:3 ratio to patients without VCF.

The medical treatment history of all enrolled patients was reviewed. None of them had undergone chemotherapy or osteoporosis treatment before or during the study. The follow-up MRI was performed 4.5 ± 2.4 months after the baseline MRI. The occurrence of a VCF was determined in an additional MRI 13.0 ± 8.5 months after baseline. The CT examinations were performed within 1 month of the baseline MR imaging.

### 2.2 Magnetic resonance imaging and measurements

MR images, including the lumbar spine, were acquired by two 3T-MRI systems (both Elition, Phillips Healthcare and Ingenia, Phillips Healthcare). Patients were placed in a supine position with a 16-channel anterior torso coil array and an inbuilt posterior 12-channel coil array. The imaging sessions included CSE-MRI for PDFF and T2* measurement, axial and coronal T2-TSE sequences, as well as axially acquired T1-weighted sequences with spectral fat saturation with and without contrast administration. For PDFF and T2* measurements, an axial six-echo 3D multi-echo gradient-echo sequence was employed, capturing all echoes in a single TR using bipolar gradients. The imaging parameters were as follows: repetition time (TR)/first echo time (TE1)/echo time step (ΔTE) = 7.8/1.35/1.1ms, field of view (FOV) = 300 x 400 x 150 mm³, acquisition voxel size = 2 x 3 x 6 mm³, reconstruction voxel size = 1.13 x 1.13 x 6 mm³, receiver bandwidth = 1678 Hz/pixel, frequency direction = anterior/posterior (A/P), 1 average, scan time = 9.3 s. To minimize T1 bias effects, a flip angle of 3° was utilized (35). Complex multi-echo gradient-echo images were processed using the fat quantification routine provided by the vendor (mDixon Quant, Philips Healthcare). After phase correction, a complex-based water-fat decomposition was performed, incorporating a single T2* correction and a pre-calibrated fat spectrum considering the multiple peaks in the fat spectrum. A seven-peak fat spectrum model was employed. PDFF maps were computed as the ratio of fat signal over the sum of fat and water signals, and PDFF and T2* maps were extracted (36, 37). Besides PDFF maps, T2* maps were utilized to assess the muscle and bone composition of the participants.

**Figure 1:**
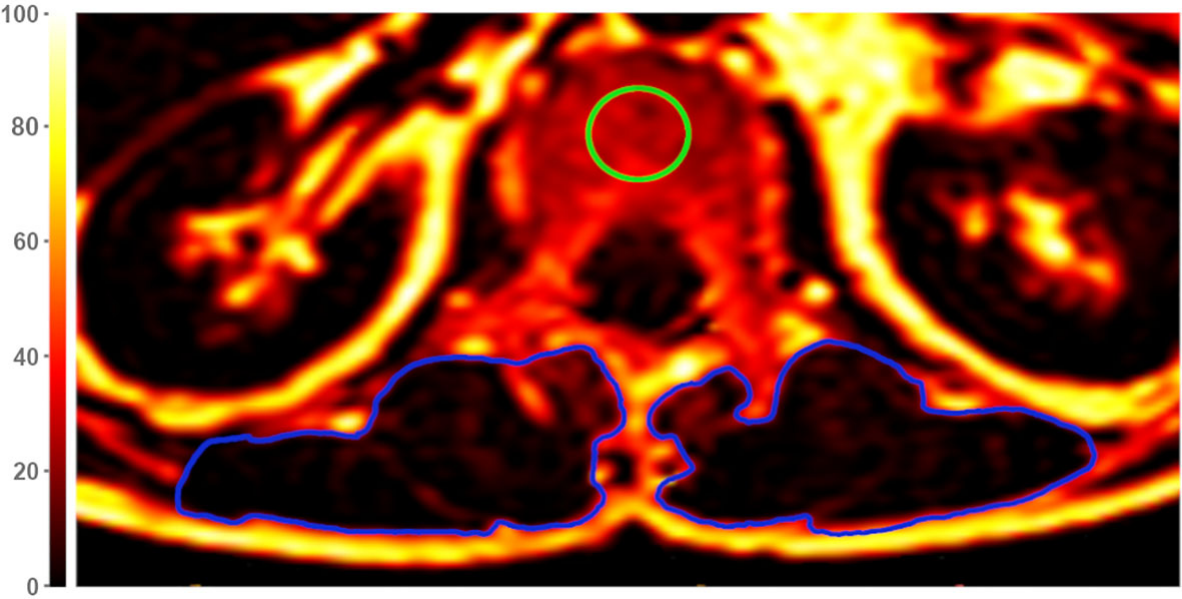
Exemplary axial Proton Density Fat Fraction (PDFF)-map at height of lumbar vertebra 1 (L1). For measurements of vertebral PDFF and T2*, regions of interest (ROIs) were placed in the center of each vertebra (green circle). Muscle PDFF, T2*, and cross-sectional area (CSA) were measured by segmentation of the paraspinal muscle (PSM) on both sides (blue circles). The color scale indicates PDFF values in [%].

Segmentation of the thoracolumbar vertebral bone marrow and PSM were performed manually by F.T.G. and Y.L. (3 and 4 years of experience in musculoskeletal imaging) on the PDFF and T2* maps using the IDS7 PACS (Sectra AB, Linkoeping, Sweden). The fractures were classified by two board-certified radiologists (B.J.S. and A.S.G., both with 12 years of experience in musculoskeletal imaging) by evaluating the involvement of the posterior column, superior and/or inferior endplate and deformity (crush, biconcave, wedge) of the vertebra. Fractures were classified with the Genant classification (38). B.J.S. and A.S.G. also ensured, in conjunction with the clinical history, that no morphological indications of malignant fractures were present in any of the sequences. Cylindrical ROIs were placed in axial PDFF and T2* maps in the center of the thoracic vertebrae (T) 11 to the lumbar vertebrae (L) 4, and the mean PDFF and T2* values were obtained for each vertebra (**Figure 1**). No ROIs were placed into fractured vertebrae at the six months follow-up. Beginning at T11, five consecutive slices on both sides were segmented in the PSM as illustrated in **Figure 1** and values were averaged. Furthermore, the CSA values of each slice were noted for both sides and averaged. The longitudinal change of PDFF, T2* and CSA in the PSM and the change of PDFF and T2* in the vertebral bone marrow was calculated as the difference between the follow-up and the baseline values.

To evaluate the intrareader reproducibility of PDFF, T2*, and BMD values, a random sample of 10 subjects was selected and reanalyzed after 8 weeks by the same radiologists Y.L. and G.C.F. F.T.G. independently analyzed a random sample of 10 subjects after a 6 months interval from the initial review to evaluate inter-reader reproducibility.

### 2.3 Computed tomography and BMD measurement

All included patients received CT images using a dual-layer dual-energy CT (IQon Spectral CT, Phillips Healthcare, Amsterdam) or a multi-slice detector CT (MDCT) (Phillips iCT 256, Phillips Healthcare). The patients were positioned in supine position and scans obtained in craniocaudal direction. The scanning parameters followed routine clinical protocols: collimation of 0.9 mm, pixel spacing of 0.4/0.3 mm, pitch factor of 0.8/0.9, tube voltage of 120 kV, and a modulated tube current ranging from 125 to 250 mAs.

The trabecular bone of the entire vertebral bodies from L1 to L4 was manually segmented using the IDS7 PACS (Sectra AB, Linkoeping, Sweden) by a radiologists G.C.F. in the axial plane, excluding cortical bone. The mean Hounsfield Unit (HU) value for each non-fractured vertebra was calculated, and the average HU value for each patient was determined by averaging the mean HU values of the vertebrae. Fractured or degenerative altered vertebrae (i.e., vertebrae exhibiting osteoarthritic changes such as osteophytes, endplate sclerosis, reduced vertebral height, or vertebrae with vertebra-/kyphoplasty) were excluded from HU measurement. The HU units were used in a previously described and tested conversion equation to calculate the BMD of the lumbar vertebrae: 0.928 g/cm³ × HU + 4.5 g/cm 3 for the IQon Spectral CT and 0.855 g/cm³ × HU + 1.172 g/cm³ for the Philips iCT 256 (39). Osteoporosis was defined as a BMD less than 80 mg/cm³, while osteopenia was defined as a BMD ranging from 80 to 120 mg/cm³ (40).

### 2.4 Statistical analysis

Statistical analysis was performed by Y.S. with RStudio Build 764 and R version 4.4.0 (R Foundation for Statistical Computing, Vienna, Austria). All tests were performed with a two-sided significance level of α = 0.05. Metric variables are presented as mean ± standard deviation. Shapiro-Wilk test was used to assess the distribution of data. Group comparisons for normally distributed metric variables were assessed for equal variances using the Bartlett Test. If variances were equal, the two-sample t-test was utilized; otherwise, the Welch test was employed. For non-normally distributed metric data, equal variances were examined with the Fligner-Killeen Test. With equal variances, the Wilcoxon rank sum exact test/Mann-Whitney U test was applied; otherwise, Mood’s median test was used for group comparisons. Categorial data was compared using Fisher’s Exact test if the sample size in a group was less than 5, otherwise the Chi-squared test was performed. In order to distinguish patients with and without VCFs, ROC curves were generated to evaluate the longitudinal PDFF cutoff values of the vertebral bone marrow and PSM based on sensitivity and specificity. For each ROC curve the optimal cutoff value with the highest Youden Index was selected. In addition to calculating the AUC, we determined the 95% confidence interval with p-value. Multivariable logistic and linear regression models were calculated to explore the association between VCFs and PDFF by adjusting for sex, age and BMD.

Intra- and inter-reader reproducibility of T2*, PDFF, CSA and BMD values were evaluated by computing the intraclass correlation coefficient (ICC) and the root mean square coefficient of variation (RMSCV) of the difference between the measurements performed by the readers.

## 3 Results

**Figure 2:**
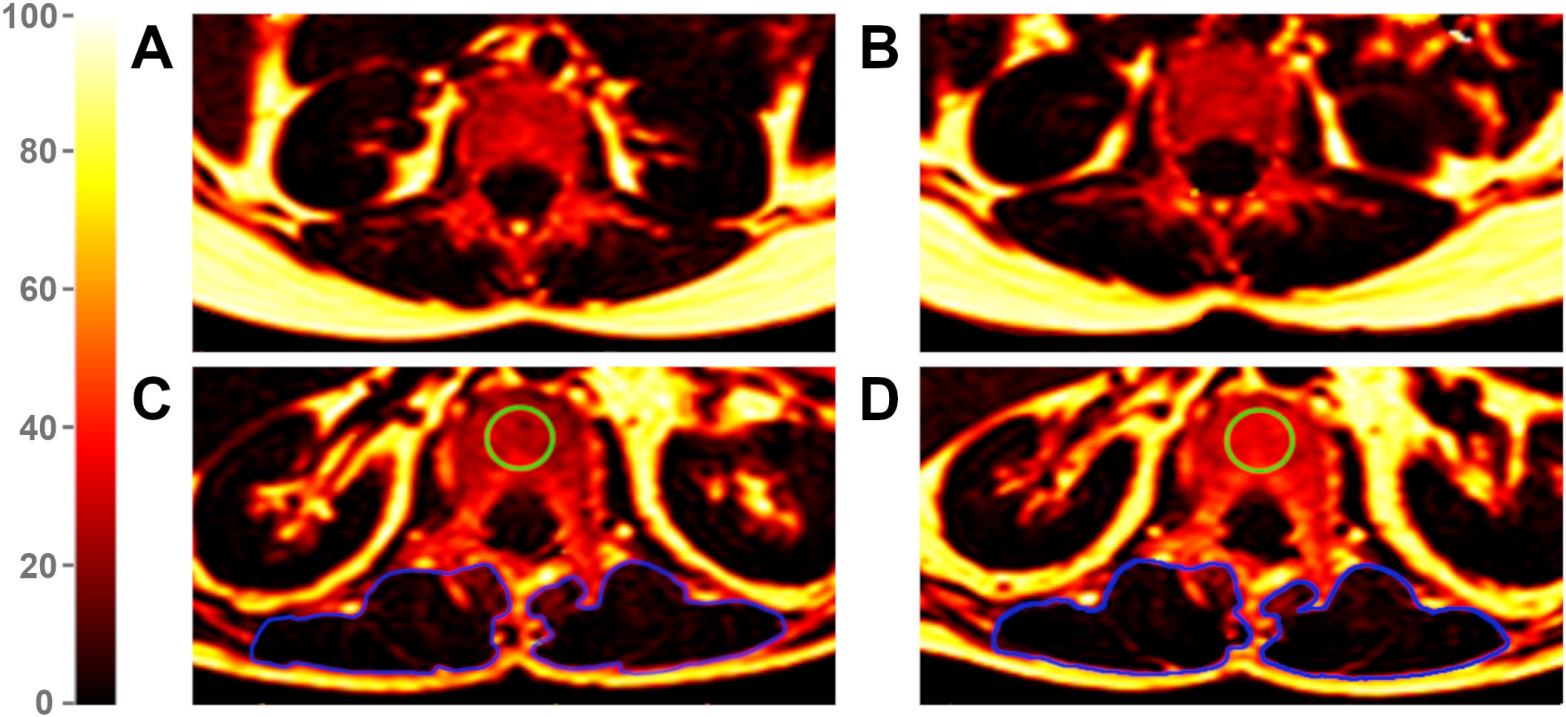
Proton Density Fat Fraction (PDFF) maps of a patient without vertebral compression fracture (VCF) at baseline **(A)** and follow-up **(B)** and of a patient with VCF at baseline **(C)** and follow-up **(D)**. The green region of interest (ROI) illustrates an exemplary measurement of the vertebral bone marrow in a VCF patient, visually highlighting an increase in fat infiltration from baseline **(C)** to follow-up **(D)**. The blue ROIs mark the paraspinal muscles. Due to an average PDFF change of 2.4% in PSM from baseline **(C)** to follow-up **(D)** among VCF patients, changes in color remain subtle. The color scale indicates PDFF values in [%].

Fourteen patients (66.8 ± 7.9 years, 9 males) with incidental VCFs (**Figure 2**) were frequency matched with patients without VCFs in a 1:3 ratio (n = 42, 64.6 ± 13.3 years, 27 males). Among VCF patients, six were osteopenic and two were osteoporotic, whereas in the non-VCF group, 17 were osteopenic and two were osteoporotic at baseline. No significant association was detected between osteopenic/osteoporotic patients and the development of VCFs (p = 0.40).

**Figure 3:**
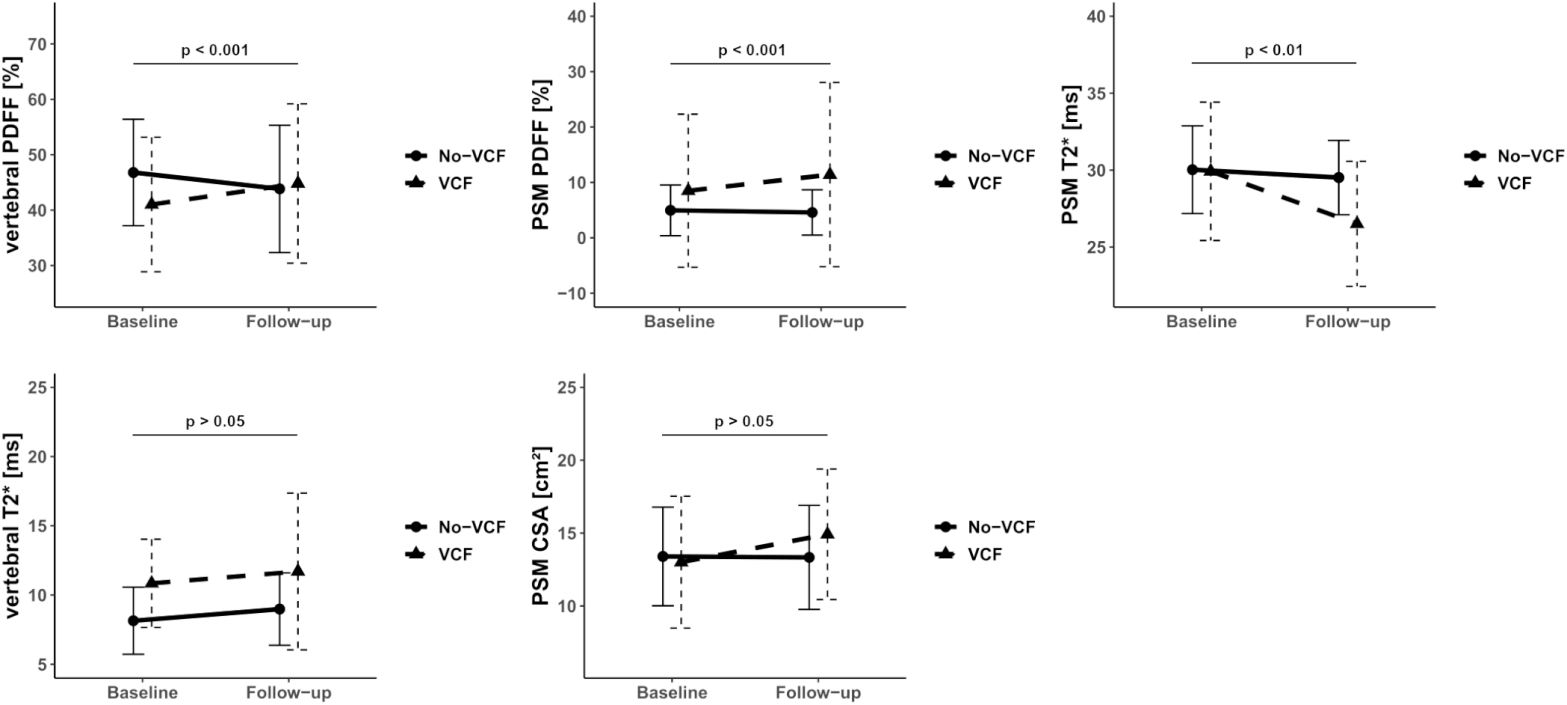
Median of Proton Density Fat Fraction (PDFF), T2* and CSA in the vertebral bone marrow and paraspinal muscle (PSM) grouped by vertebral compression fracture (VCF) status at baseline and at 6-month follow-up. The p-values refer to the change of the respective value from baseline to follow-up between patients with and without VCF.

Descriptive statistics of MRI data of PDFF, T2*, and CSA analyses for patients with and without VCFs at baseline and follow-up are listed in **Table 1**. When assessing PSM parameters, there were no significant differences between the VCF and non-VCF groups in terms of baseline PDFF (VCF/non-VCF 8.5 ± 13.8% vs. 5.0 ± 4.6%, p = 0.53). Additionally, CSA (VCF/non-VCF 13.0 ± 4.5 cm² vs. 13.4 ± 3.4 cm², p = 0.44) showed no significant difference between both groups. While the PDFF of the PSM increased significantly in patients with VCF over time, the PDFF in the non-VCF decreased significantly over 6 month (VCF/non-VCF 2.4 ± 2.8% vs.-1.0 ± 2.3%; p < 0.001, **Figure 3**; **Table 2**). The analysis of T2* relaxation time of the PSM at baseline showed no significant difference between the VCF and non-VCF group (**Table 1**). However, at follow-up, the T2* relaxation time of the PSM was significantly higher in the non-VCF group compared to the VCF group (follow-up T2* VCF/non-VCF 26.5 ± 4.1 ms vs. 29.5 ± 2.4 ms, p = 0.02). The VCF group experienced a significantly higher longitudinal decrease in T2* in the PSM over time (VCF/non-VCF -3.3 ± 2.7 ms vs. -0.4 ± 2.2 ms, p = 0.01, **Figure 3**; **Table 2**).

**Table 1:** Descriptive analyses between patients with (VCF) and without (non-VCF) vertebral compression fractures for baseline and follow-up. The values are listed as mean ± standard deviation.

	Baseline			Follow-Up		
	VCF group	Non-VCF group	*P*-value	VCF group	Non-VCF group	*P*-value
**Vertebral bone marrow PDFF of T11 - L4 (%)**	41.0 ± 12.2	46.8 ± 9.6	0.07 ^b^	44.8 ± 14.4	43.8 ± 11.5	0.83 ^b^
**PDFF of the paraspinal muscle (%)**	8.5 ± 13.8	5.0 ± 4.6	0.53 ^c^	11.4 ± 16.6	4.6 ± 4.1	**0.04 ^c^**
Vertebral bone marrow T2* of T11 - L4 (ms)	10.9 ± 3.2	8.1 ± 2.4	**0.01 ^b^**	11.7 ± 5.7	9.0 ± 2.6	0.16 ^c^
T2* of the paraspinal muscle (ms)	29.9 ± 4.5	30.0 ± 2.9	0.93 ^d^	26.5 ± 4.1	29.5 ± 2.4	**0.02 ^c^**
**CSA of the average right and left paraspinal muscle (cm²)**	13.0 ± 4.5	13.4 ± 3.4	0.44 ^c^	14.9 ± 4.5	13.3 ± 3.6	0.26 ^b^

**^a^** Mood’s median test.

**^b^** Two-sample t-test.

**^c^** Wilcoxon rank sum test/Mann-Whitney U test.

**^d^** Welch t-test.

PDFF, Proton Density Fat Fraction; VCF, vertebral compression fracture; CSA, cross-sectional area.

Statistically significant p-values (p ≤ 0.05) are highlighted in bold.

VCF group showed a significant increase in vertebral bone marrow PDFF over time (7.9 ± 7.0% vs. -2.6 ± 5.4%, p < 0.001, **Figure 3**; **Table 2**). However, no significant differences in PDFF of the vertebral bone marrow were observed between the VCF and non-VCF group, neither at baseline nor at follow-up (**Table 1**). The mean T2* value of the vertebral bone marrow was significantly higher in patients with VCFs (baseline T2* VCF/non-VCF 10.9 ± 3.2 ms vs. 8.1 ± 2.4 ms, p = 0.01), suggesting an initially slightly lower BMD. Indeed, BMD was lower among VCF patient, but the difference was not statistically significant (VCF/non-VCF 115.7 ± 37.9 mg/cm³ vs. 136.6 ± 45.6 mg/cm³, p = 0.12).

**Table 2:** Descriptive analyses for the change of Proton Density Fat Fraction (PDFF), T2* and muscle cross-sectional area (CSA) in the vertebral bone marrow and paraspinal muscle (PSM) between patients with (VCF) and without (non-VCF) vertebral compression fractures.

	VCF group	non-VCF group	P-value
**Change in PDFF over 6 months of the paraspinal muscle (%)**	2.4 ± 2.8	-1.0 ± 2.3	**<0.001** ^c^
Change in T2* over 6 months of the paraspinal muscle (ms)	-3.3 ± 2.7	-0.4 ± 2.2	**0.01** ^b^
**Change in vertebral bone marrow PDFF of T11 - L4 over 6 months (%)**	7.9 ± 7.0	-2.6 ± 5.4	**< 0.001** ^b^
Change in vertebral bone marrow T2* of T11 - L4 over 6 months (ms)	0.4 ± 3.1	0.5 ± 2.1	0.93 ^b^
**Change in CSA over 6 months of the paraspinal muscle (cm²)**	0.6 ± 6.7	-0.1 ± 1.6	0.26 ^a^

**^a^** Mood**’**s median test.

**^b^** Two-sample t-test.

**^c^** Wilcoxon rank sum test**/**Mann-Whitney U test.

**^d^** Welch t-test.

PDFF, Proton Density Fat Fraction; VCF, vertebral compression fracture; CSA, cross-sectional area. The values are listed as mean ± standard deviation.

Statistically significant p-values (p ≤ 0.05) are highlighted in bold.

Multivariable linear regression analysis, adjusted for gender, age, and BMD, revealed an average increase in PDFF of the PSM of 3.5% from baseline to follow-up (95% CI [1.8 – 5.3]; p < 0.001) in patients who developed VCF compared to patients without VCF. The change in CSA of the PSM (82.6 cm², 95% CI [-177.8 - 343.0]; p = 0.52) did not significantly differ between the groups. Multivariable logistic regression model, adjusted for gender, age, and BMD, suggested that increasing PDFF of the PSM (OR = 2.21, 95% CI [1.38 - 4.73]; p < 0.01) and vertebral bone marrow over 6 months (OR = 1.49, 95% CI [1.19 - 2.14]; p < 0.01) are significant risk factors for the development of VCF. In the same model, decreasing T2* of the PSM was also identified as a risk factor for the development of a VCF (OR = 1.89, 95% CI [1.28 - 3.33]; p < 0.01). In contrast, changes in T2* of the vertebral bone marrow (OR = 0.97, 95% CI [0.69 - 1.32]; p = 0.87) and changes in CSA of the PSM over 6 months (OR = 1.00, 95% CI [0.99 – 1.00]; p = 0.50) do not pose a risk factor for the development of VCF.

**Figure 4:**
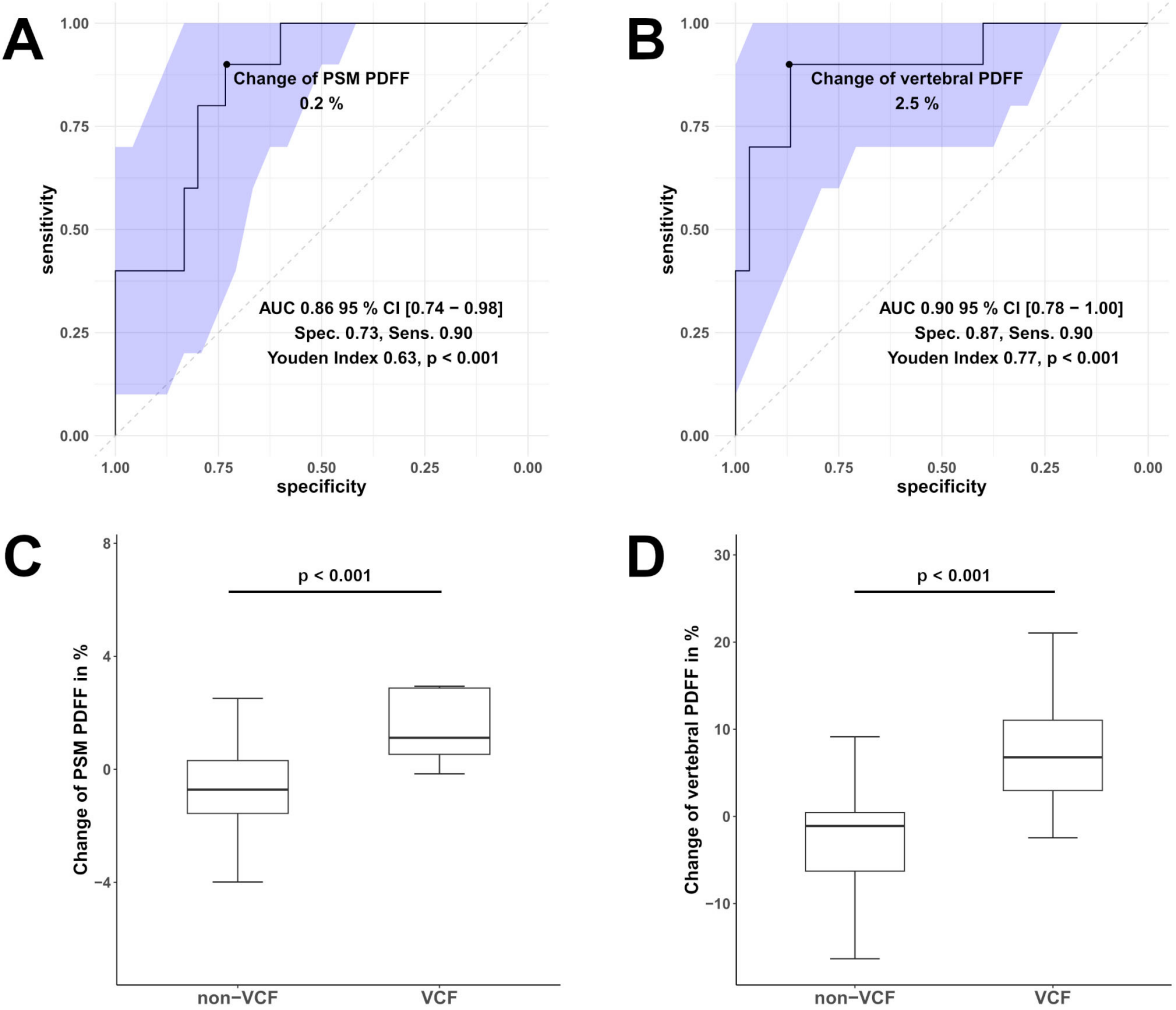
Receiver operating characteristic (ROC) curves of the change in Proton Density Fat Fraction (PDFF) in the paraspinal muscle (PSM, **(A)**) and vertebral bone marrow PDFF **(B)** were used to differentiate between patients with vertebral compression fracture (VCF) and without (non-VCF). The blue area illustrates the 95% confidence interval (CI) of the area under the curve (AUC). Additionally, boxplot **(C)** represents the median longitudinal change of PDFF in the PSM, and boxplot **(D)** shows the change of vertebral bone marrow PDFF between individuals who experienced VCF and those who did not. Respectively, the difference in in the change of PDFF PSM and vertebral bone marrow PDFF was significant (for both p < 0.001).

Moreover, ROC analysis (**Figure 4**) showed that a PDFF change of 0.2% in the PSM (AUC 0.86, 95% CI [0.74 - 0.98], specificity 0.73, sensitivity 0.90, Youden Index = 0.63, p < 0.001) can significantly differentiate VCF from non-VCF patients.

The intrareader as well as the interreader agreement for T2* (ICC for both 0.98, 95% CI [0.96 - 0.99]), PDFF (ICC for both 0.98 [95% CI, 0.96 - 0.99]), CSA (ICC for both 0.98, 95% CI [0.96 - 0.99]) and BMD (ICC intrareader 0.99, 95% CI [0.98 – 0.99] and interreader 0.99, 95% CI [0.97–0.99]) measurements were excellent. Intra- and interrater reproducibility, assessed by calculating the RMSCV, also demonstrated excellent agreement for T2* (RMSCV intrarater 0.8% and interrater 0.9%), PDFF (RMSCV intrarater 0.9% and interrater 0.8%), CSA (RMSCV intrarater 0.7% and interrater 0.8%) and BMD (RMSCV intrarater 0.4% and interrater 0.5%) measurements.

## 4 Discussion

This study assessed the associations between the occurrence of incidental VCFs and the longitudinal changes in PDFF, T2* and CSA of the PSM and vertebral bone marrow in the thoracolumbar region. Although no significant baseline difference in BMD was observed, the increase in PDFF in the PSM and vertebral bone marrow was significantly higher in patients who developed a VCF compared to those without VCF.

The marked increase in PSM PDFF within six months indicates a rapid fatty degeneration, potentially linked to the occurrence of VCFs. The CSA of the PSM showed a marginal increase in these patients, however this change did not reach statistical significance. Several studies have investigated the impact of elevated muscle PDFF on both muscle function and bone health. Fatty infiltration of the PSM has been associated with severe back pain and structural abnormalities in the lumbar spine, such as reduced disc height and decreased muscle strength (31, 41). In relation to bone health, the PDFF of the erector spinae, multifidus, and psoas muscles has been shown to be significantly higher in osteopenic patients (42).

Our findings, consistent with previously published studies, demonstrated an increase in PDFF within the vertebral bone marrow among individuals with VCFs, whereas BMD showed no significant differences. Although an inverse correlation between vertebral bone marrow PDFF and BMD has been well established (17–21), a recent study reported an increase in PDFF in vertebral bone marrow prior to incidental fractures, while BMD remained unchanged. This finding underscores the potential predictive value of PDFF over BMD in forecasting the occurrence of VCFs (25). Additionally, elevated fat content in the vertebral bone marrow has been linked to reduced bone quality and the presence of osteoporosis (29, 43). Postmenopausal women, a population at higher risk for osteoporotic vertebral fractures, exhibited significantly higher PDFF in the vertebral bone marrow compared to premenopausal women (44). Furthermore, insulin resistance is associated with increased vertebral bone marrow PDFF and a heightened risk of fractures. This suggests fatty vertebral infiltration may serve as an additional risk factor for fractures in patients with pathological glucose metabolism (45–47).

Age, physical inactivity, muscle atrophy, and reduced BMD are known risk factors for VCFs (6, 8–11). These factors are also linked to fatty infiltration in bone and muscle (18–21, 31, 48, 49). Muscle atrophy weakens spinal stability, leading to more mechanical stress and eventually, results in inactivity (50). We hypothesize that these factors interact in a vicious cycle: muscle atrophy reduces physical activity, promoting fatty muscle infiltration, thereby further reinforcing inactivity. In addition, muscle communicates with bone through various endocrinologic pathways influencing bone turnover and, consequently, BMD (7, 51). In our study, regression models demonstrated that PDFF of the PSM and vertebral bone marrow were significantly elevated in VCF patients, independent of age, gender, and baseline BMD. Unfortunately, no data on physical activity is available, which limits our ability to fully explore its role in the observed findings. Including physical activity metrics in future studies could provide valuable insights into the interplay between muscle function and fracture risk.

To the best of our knowledge, a decrease in T2* in skeletal muscle has not yet been described in patients with VCF or osteoporosis. T2* values are influenced by factors such as metabolic state, blood volume, ischemia, and physical activity (52–55). A T2* decrease reflects magnetic field inhomogeneity. In older adults muscle tissue accumulates dense bodies containing iron and lipids, causing disturbance in the magnetic field (56). In the VCF group, increased fatty infiltration in the PSM may have contributed to greater magnetic field inhomogeneities, measurable as T2* decay.

No significant differences were found in BMD and vertebral bone marrow T2* values between the VCF and non-VCF groups, which reflects the fact that both parameters are primarily surrogates for the calcified trabecular bone components. These measures may not necessarily detect subtle changes indicative of bone health deterioration, ultimately leading to VCFs. As PDFF of both vertebral bone marrow and muscle successfully differentiated between the two groups in our study, these parameters may represent more reliable predictors of bone pathologies. On a microscopic level, bone consists of bone trabeculae and bone marrow. These two tissue types create inhomogeneities in the magnetic field, which also result in the shortening of the effective transverse relaxation time, detectable through T2* measurements (57, 58). T2* of vertebral bone marrow has been shown to negatively correlate with BMD. This suggests that a reduction in bone trabeculae increases magnetic field homogeneity within the vertebral bodies, resulting in longer T2* times (59). This correlation has been confirmed at the microstructural level using T2* mapping, where higher T2* values were associated with reduced trabecular density and increased trabecular spacing, indicating greater bone fragility (32).

An important limitation of this study is the small sample size of patients with VCFs. Hence, certain statistically significant differences may not have been detected. Another limitation of this study is the low resolution of the PDFF and T2* maps, which was necessary in order to cover a large field of view in both groups. Notably, T2* mapping within skeletal muscle might be more accurately estimated with higher spatial resolution. The low resolution employed in this study may account for the observed T2* decay in PSM among VCF patients as PDFF increases, potentially due to increased averaging of field inhomogeneity effects. The segmentations of the maps were time consuming, however the assessment of PSM through automated segmentations could be facilitated in the future by using automatic deep learning techniques (60, 61). Additionally, a longer follow-up period may reveal more pronounced changes in e.g. CSA or age, providing further insight into their role in VCF development and potentially revealing their predictive value. Lastly, PDFF changes may have emerged as a consequence of factors such as pain-related muscle inactivity and fat atrophy following a fracture, limiting their predictive validity.

In conclusion, the PDFF of the PSM increased over a 6 month period in patients with VCFs. This change was also detected within the vertebral bone marrow, which is consistent with previous studies. ROC modeling revealed an excellent discrimination between VCF development when choosing a cutoff value of 0.23% for the change in PDFF PSM. Our findings suggest that the longitudinal assessment of PDFF of the PSM and vertebral bone marrow may serve as useful indicator for musculoskeletal health and may enable the prediction of incidental vertebral compression fractures.

## Data Availability

The raw data supporting the conclusions of this article will be made available by the authors, without undue reservation.
